# E-Health Psychological Intervention for COVID-19 Healthcare Workers: Protocol for its Implementation and Evaluation

**DOI:** 10.3390/ijerph191912749

**Published:** 2022-10-05

**Authors:** Alejandro Dominguez-Rodriguez, Reyna Jazmín Martínez-Arriaga, Paulina Erika Herdoiza-Arroyo, Eduardo Bautista-Valerio, Anabel de la Rosa-Gómez, Rosa Olimpia Castellanos Vargas, Laura Lacomba-Trejo, Joaquín Mateu-Mollá, Miriam de Jesús Lupercio Ramírez, Jairo Alejandro Figueroa González, Flor Rocío Ramírez Martínez

**Affiliations:** 1 Health Sciences Area, Valencian International University, 46002 Valencia, Spain; 2 Departamento de Clínicas de Salud Mental, Centro Universitario de Ciencias de la Salud, Universidad de Guadalajara, Guadalajara 44340, Mexico; 3 School of Psychology, Universidad Internacional del Ecuador, Quito 170201, Ecuador; 4 Facultad de Estudios Superiores Iztacala, Universidad Nacional Autónoma de México, Mexico City 54090, Mexico; 5 Departamento de Ciencias de la Salud, Universidad Autónoma de Ciudad Juárez, Juarez City 32300, Mexico; 6 Department of Personality, Evaluation and Psychological Treatment, Universitat de València, 46010 Valencia, Spain; 7 Maestría en Psicología de la Salud, Centro Universitario de Ciencias de la Salud, Universidad de Guadalajara, Jalisco 44340, Mexico; 8 Rectoría, Universidad Autónoma de Ciudad Juárez, Juarez City 32410, Mexico

**Keywords:** COVID-19, internet-based intervention, mental health, healthcare workers, anxiety, depression

## Abstract

(1) Background: Healthcare workers have been affected by the COVID-19 pandemic. Digital interventions have been carried out that have been effective with this population; however, few have been reported in Latin America. Our aim is to describe the components and methods to evaluate the feasibility and utility of an online multi-component psychological intervention for healthcare workers in Mexico during COVID-19. (2) Methods: This study is a randomized clinical trial with two arms: (1) self-applied intervention and (2) intervention delivered online by therapists. The participants are randomly assigned to one arm, receiving the same treatment contents in both groups. The “Personal COVID” intervention consists of an internet platform containing 9 nuclear and 3 complementary modules. The objectives of the intervention are: (1) to reduce anxiety, depressive symptoms, burnout, and compassion fatigue, and (2) to increase the quality of life, sleep quality, self-care, and their skills to give bad news. The protocol has been registered on ClinicalTrials.gov (identifier: NCT04890665). (3) Discussion: This protocol is designed according to the highest scientific standards following the SPIRIT guidelines. The “Personal COVID” intervention is expected to be of high efficacy in treating the emotional distress of healthcare workers and promoting their health during the COVID-19 pandemic.

## 1. Introduction

The COVID-19 pandemic has brought a series of effects on the physical and mental health of healthcare workers [1]. It has been found that some of the most frequent consequences in healthcare workers who care for patients with COVID-19 are anxiety, depression, insomnia, stress, and compassion fatigue [2,3,4,5,6]. Since healthcare workers have been under stressful conditions for more than two years in the face of the current pandemic, their long-term mental health has been affected. Some healthcare workers are experiencing high levels of burnout, anxiety, anguish, anger states, post-traumatic stress, and depression [7,8,9,10].

Some risk factors for the healthcare workers coping with these psychological disorders are: being in contact with infected patients, loss of friends or loved ones due to COVID-19, excessive workload, and neglecting even basic activities [11,12]. Regarding the insomnia, some of the correlates are occupational conditions (i.e., work at high-risk environment, having greater uncertainty about the management of patients with COVID-19), sociodemographic characteristics (i.e., being a woman, middle-aged, lower levels of education), and social factors (living with a person with a chronic disease, receiving negative comments about their work from family and friends, and poor social support) [11].

In developed countries the data about the impact on mental health workers is abundant; however, in low-income and middle-income countries, such as Mexico, the data is scarce. Regarding the data for Mexico, the most common psychological consequences reported are insomnia, depression, and post-traumatic stress disorder, with physicians and paramedics the most affected [12,13]. In a study that evaluated the psychological impact on 462 Mexican nurses, the researchers identified that 46.72% of them had traumatic stress responses from moderate to severe, and that 42.40% of participants exhibited a high level of emotional exhaustion [14]. A systematic review and meta-analysis identified that more than a fifth of nurses in professional practice during COVID-19 presented depression and almost a third, anxiety [15]. It has also been reported that physicians who were in contact with infected patients with COVID-19, felt at a higher risk of getting infected and infecting their families with the virus, causing them moderate to high stress [13]. The study of Robles-Pérez et al. [16] confirmed that the frontline COVID-19 team workers in Mexico were at excessive risk of getting infected by SARS-CoV-2, and this risk was higher for respiratory therapists, nurses, and patient transporters.

Currently, due to the impact of the COVID-19 pandemic on the mental health of healthcare workers, digital psychological interventions have been developed to improve organizational, peer support and personal strategies [17,18]. The literature highlights that both digital and non-digital psychological interventions have been aimed at both formal caregivers (health professionals who work inside or outside a hospital; namely, physicians, nurses, technicians, or administrative staff) [17,18] and informal (caregivers providing support to a sick or disabled person) [19]. It has also been found that other intervention programs have targeted a broader population: paramedics, firefighters, community health workers, social service providers, military forces, police officers, forensic workers, and other non-professionals trained to respond to emergency or disaster situations [20].

Serrano-Ripoll et al. [21] developed a mobile application with topics such as emotional skills, lifestyle behavior, stress and burnout, and social support aimed at healthcare workers. In addition, Blacke et al. [22] developed and evaluated a digital learning resource aimed at promoting and supporting psychological well-being in health workers in the United Kingdom. The digital tools were highly used by health workers, and also showed positive results in relation to practicality or utility, resource challenges (low cost), attitudes, acceptability, and usability [23]. Likewise, De Kock et al. [18] conducted a randomized controlled trial in which they evaluated the use of two mobile applications versus a waiting list condition to reduce depression and anxiety, and increase mental well-being, mental toughness, and gratitude; the results showed improvements in the groups that received the interventions compared to the control group (waiting list).

Furthermore, a systematic review identified three studies from China and two from Italy that documented the efficacy of digital psychological support interventions aimed to healthcare providers and informal caregivers during COVID-19. The findings reported that the interventions helped to reduce anguish, anxiety, depression, and exhaustion, as well as promote positive emotions, teamwork efficiency, improve well-being, quality of life and self-efficacy. Likewise, these interventions were delivered by different ways such as chat, email, playlist by mobile phone or by videoconference (telemedicine) [19]. However, within these studies, three opted for a single group pre-post design and two a pre-post comparative design; within the comparative designs, one was aimed at caregivers of young people with or without eating disorders, and in another, telehealth provided by videoconference and by telephone calls was compared. In this last study it was reported that telemedicine by videoconference was associated with a greater resilience and well-being for both people with neurocognitive disorder and their caregivers at home, compared to telemedicine by phone calls [24]. In another systematic review, early psychological interventions were identified to address the psychological impact of COVID-19 on healthcare workers; whilst these interventions were not implemented digitally, they showed useful results both in reducing the emotional impact of psychological distress (e.g., general psychopathology, post-traumatic stress disorder and stress) and in promoting positive psychological domains (e.g., resilience, self-efficacy and life satisfaction) [20]. Similarly, another systematic review identified various digital and non-digital interventions to address mental health problems in healthcare workers during infectious disease outbreaks [25]. The results identified four categories of interventions: (1) informational support (training, guidelines, prevention programs), (2) instrumental support (personal protective equipment, protection protocols), (3) organizational support (manpower allocation, working hours, provision of rest areas) and (4) emotional and psychological support (psychoeducation, mental health support team, peer-support and counseling, therapy, digital platforms and tele-support) [17].

In addition, a survey carried out in different Spanish hospitals sought to describe the main characteristics and components of the psychological intervention programs that had been implemented for healthcare workers exposed to COVID-19 patients. The application of both face-to-face and online interventions (individual or group), focused mainly on emotional regulation (reduction of anxiety and stress), reduction of physiological arousal, and improving communication skills between professionals and patients [26]. The most prevalent contents were psychoeducation and mindfulness, with cognitive-behavioral therapy (CBT) the most used in individual interventions [19]. In accordance, there is currently no standardized psychological intervention model aimed at healthcare workers in situations of infectious diseases; however, the available digital interventions have been directed towards reducing emotional distress and promoting positive aspects [20]. Approaches such as CBT [19,21,24,25,26,27], behavioral activation (BA) [17], acceptance and commitment therapy (ACT) [28], mindfulness, yoga, music therapy and progressive relaxation [21,22,24,25], resilience [17,20,22,24], self-care, social support [22], as well as positive psychology (PP) techniques to increase mental well-being, mental toughness and gratitude [18] have been applied for healthcare workers during the COVID-19 pandemic. In addition, the systematic reviews identified that part of the objectives of the interventions were to promote well-being, quality of life and self-efficacy [19], using: psychological first aid (PFA); eye movement desensitization and reprocessing (EMDR); anticipate, plan, and deter (APD); resilience at work (RAW); resilience and coping for the healthcare community (RCHC) and trauma risk management (TRiM)) [20]. Further, the application to health care workers of the Health Belief Model (HBM) has been suggested, in order to promote healthy behaviors (mitigate disease threats) and modify those behaviors that generate emotional distress [29].

In this context, psychological digital interventions may be the best option to overcome barriers in mental healthcare for this population [25]. These interventions can be adapted to different media and serve different groups, and they should always be based on scientific evidence [17,30]. Despite the benefits of online interventions, there are areas of opportunity in this field that need further studies, such as improving adherence. The dropout rate in non-guided internet-based treatments are usually higher compared to the guided version [31]. Mellville et al. published in 2010 a literature review regarding the dropout rates on online interventions, raising awareness of the needs of working on this area; no significant improvements have been achieved in this sense [32]. Therefore, further research must be conducted regarding this issue. A possibility way to increase adherence to these interventions is to improve the interaction between users and the system. In line with this, an option is to analyze a sparsely studied area on psychological treatments by the internet in terms of the user experience (UX). The UX approach refers to the experience that a user has with a product, with special emphasis on human-product interaction [33,34]. The World Health Organization has declared the need to develop evidence-based self-help interventions for healthcare workers to protect their mental health [30]. 

An aspect to consider in research into psychological online interventions is that the feasibility and potential benefits of internet-based interventions are much less known in low- or middle-income countries, such as Mexico; only a few studies have tested the effectiveness of these interventions through randomized clinical trials designs in these settings [35]. There is still a lack of these studies on digital interventions aimed at healthcare workers comparing digital interventions versus teletherapy delivered by videoconference [17,19,28]. There are several studies that document the efficacy of therapist-directed (synchronous through videoconference) and self-directed (asynchronous through digital interventions) treatments for various types of mental health problems in different populations. Nonetheless, the literature reports that some digital treatment modalities that are asynchronous are less effective than synchronous ones [28]. In this sense, in the context of healthcare workers facing COVID-19, these comparisons between modalities have not been explored. Despite the above, in Mexico, psychological digital interventions have been a good option in the current pandemic, since the users are not exposed to the risk of contagion [36]. Another relevant aspect is the cost efficacy of online interventions, especially in low- and middle-income countries where the available budget is scarce for mental health treatments. The Pan American Health Organization (PAHO) highlights that median spending in mental health services stands globally at 2.8% of total government health spending and in the Americas, spending ranges from 0.2% to 8.6% [37]. The systematic review of Arnberg et al. [38] analyzed the cost-effectiveness of psychological online interventions, citing a study [39] which concluded that internet-delivered cognitive behavioral therapy had a cost per quality-adjusted life year of £20 000compared to treatment as usual. 

In summary, it is key to develop a digital platform that integrates psychological components that allow healthcare workers on the frontline of COVID-19 to increase their physical and emotional well-being and that at the same time are cost-effective, especially for countries with a limited budget to provide psychological treatments to the population. It is important to develop coping strategies that help mitigate the impact of the pandemic and at the same time to expand the probabilities of adherence to the treatment.

### Aims and Hypothesis

Given the mental health needs of healthcare workers, the “Personal COVID” platform was created. The objectives of the intervention are: (1) to reduce anxiety, depressive symptoms, burnout, and compassion fatigue, (2) to increase the quality of life, sleep quality, resilience, and self-care. Following the UX principles, it is expected that the dropout rates will be low, and the system will be evaluated as easy to use.

Thus, the goal of this trial is to evaluate the effectiveness of an online psychological intervention for healthcare workers in Mexico, during the COVID-19 pandemic. To comply with ethical considerations, any participant who fulfills the inclusion criteria will receive a treatment, and therefore two groups have been designed: (1) self-applied intervention (experimental group), and (2) same intervention applied by a psychotherapist via online treatment (active control group).

The hypothesis to be tested is that the online psychological intervention “Personal COVID” for healthcare workers will reduce anxiety, depression, and burnout as well as compassion fatigue, and will improve quality of life, sleep quality, resilience, and self-care. 

## 2. Materials and Methods

### 2.1. Study Design

**Figure 1:**
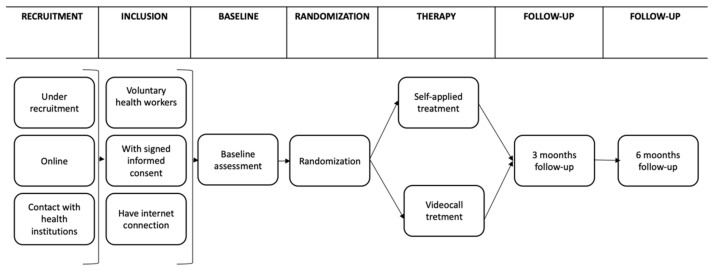
Study design.

A randomized controlled clinical trial with two independent groups will be used. Measurements will be made at four stages of the study: pretest, post-test, follow-up at 3 months and follow-up at 6 months (Figure 1). Participants will be randomly assigned to one of two groups:

(a) Experimental group, “Personal COVID” intervention, that consists of nine online nuclear sessions and three complementary. Each session will be delivered every third day for the participant to perform the tasks. An email will be sent between each session, in which the summary of the next session will be provided. The experimental group will receive the intervention in a self-applied modality, through the “Personal COVID” platform.
(b) The active control group, will receive the same intervention but with a therapist, through videoconference. The number of sessions and the content as well as the periodicity, will be the same as the experimental group. The intervention will be delivered by psychotherapists from the University of Guadalajara and the National Autonomous University of Mexico through online treatment.

This study protocol follows the SPIRIT guidelines (Table S1, Supplementary Materials), and it was registered in the Ethics Committee in the Research of Autonomous University of Juarez City (CEI-2021-1-266) and was registered in Clinical Trials (NCT04890665).

### 2.2. Participants and Eligibility Criteria

#### 2.2.1. Sample Size

Voluntary healthcare workers will participate in the intervention. The total sample will be at least composed of 49 participants (24 in active control group and 25 in experimental group), based on previous interventions administered online [36], as well as on the potency analysis (1 − β = 0.95, α = 0.05, Cohen’s d = 0.80), considering that values equal or greater than 0.80 in Cohen’s d are considered large effect sizes [40]. The above analysis was done with GPower and revealed the need to consider at least 36 participants. We will include 13 more participants based on possible experimental mortality, which has been reported to be around 36%.

#### 2.2.2. Inclusion Criteria

Voluntary healthcare workers, who have signed the informed consent, and have internet access via mobile, tablet or computer, will be included in the treatment.

#### 2.2.3. Exclusion Criteria

The exclusion criteria exclude healthcare workers who at the time of the evaluation are engaging: (1) in active substance use, (2) in psychotherapeutic treatment, (3) in psychopharmacological treatment, (4) present a diagnosed mental health disorder, (5) obtain in the enrolment evaluation a severe depressive disorder or post-traumatic stress disorder diagnosis, and (6) report active suicidal ideation or suicide attempt (in the last three months). The participants that meet the exclusion criteria will not have access to the platform; however, they will be referred to specialized helplines.

### 2.3. Randomization Process and Blinding

**Figure 2:**
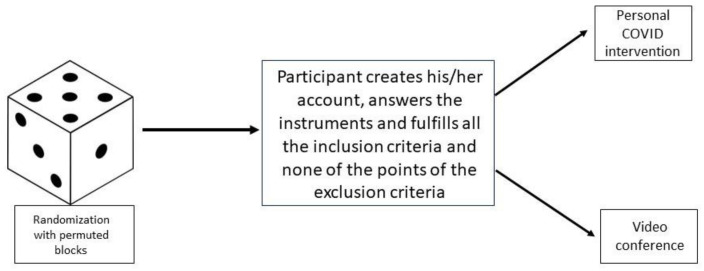
Randomization process.

The Study Randomizer software [41] will be used. The randomization procedure will use a permuted blocks algorithm through which a member of the project (PEHA) will obtain the location of the participants before they join the intervention. After the creation of an account on the platform by the participant, and its evaluation, the platform will assess the fulfillment of all the inclusion criteria and the absence of exclusion criteria, then the participant will be assigned to the corresponding group (Figure 2). All the persons who consented to participate and meet the eligibility criteria will be randomly assigned to Experimental Group and Active Control Group, controlling sociodemographic variables in order to homogenize the sample characteristics and to avoid potential biases. Participants of the Experimental Group and Active Control Group will not be blinded to the intervention, because of the specific features of the eHealth program. However, the investigators who analyze the data will be blinded to the group allocation.

### 2.4. Procedure and Recruitment

#### 2.4.1. Screening Assessment

The “Personal COVID” platform was available for the healthcare workers on 16 July 2021 and is still available in www.personalcovid.com, accessed on 24 September 2022; this platform is part of one of the developments of ITLAS group (Internet Treatment for Latin American and Spain) [42]. The recruitment process will be through social networks in order to advertise the initiative among potentially interested healthcare workers, with the collaboration of the Instituto Nacional de Psiquiatría Ramón de la Fuente Muñiz. Participants who sign the informed consent will be given access to answer the questionnaires. The questionnaires to evaluate the eligibility of the participants will be the Plutchik Suicide Risk Scale (PSRS), the Post-Traumatic Stress Scale (PTSD) and the Center for Epidemiologic Studies Depression Scale, revised version (CES-D-R). Once they complete the questionnaires, they will receive an email indicating that they will be given access once it is verified that they meet the eligibility criteria.

#### 2.4.2. Baseline Assessment

The questionnaires to evaluate the primary outcomes will be applied in the platform before accessing the intervention.

#### 2.4.3. Access to Intervention and Follow-Up Assessment

All volunteer participants who meet the eligibility criteria, who agree to participate in the intervention and who answer the questionnaires, will be included in the intervention. Once they answer the questionnaires, which evaluate the primary and secondary outcomes (pretest), both the experimental group and the active control group will receive the intervention of nine sessions with the option of three complementary sessions, with a periodicity of one session every three days. All the participants will be allowed to log in to the virtual platform using their registered e-mail. The evaluation tests will be administered after the intervention (post-test); then, a follow-up will be carried out at three and six months, to evaluate the maintenance (Table 1).

### 2.5. “Personal COVID” Intervention

#### 2.5.1. Design of the “Personal COVID” Intervention

This intervention was designed through the principles of UX, in order to increase the probability that the platform will succeed in meeting the requirements and be perceived as easy to use, attractive and useful by the participants. This process starts with a problem that affected the population, in this case, the emotional impact on healthcare workers. Possible solutions are taught about the posed problem; for this reason, a search was made in the different databases to collect information on other interventions already available and if possible, to access them. The UX process was conducted by the main author who is a certified UX designer, along with two students (JAFG, MJLR) and a UX researcher who provided support. The steps that were followed for the UX process were: (1) Health care workers were interviewed regarding the impact of the COVID-19 pandemic on their mental health and possible suggestions for an online intervention such as “Personal COVID”. (2) Based on the information collected in the semi-structured in-depth interviews, a survey was carried out which was sent to healthcare workers which explored similar questions to those in step one but with a broader population. (3) After analyzing the recordings of the interviews and the survey results, affinity mappings were conducted to find similar requests, needs or suggestions from the participants toward the platform. (4) User Personas were created from the results; these are fictional characters based on the overall results of the participants’ interviews [43]. (5) Afterwards, User Journey Maps and User Flows were also created, along with a site map proposal. (6) In addition, a card sorting test was distributed online through Optimal Workshop [44], (7) Wireframes with the proposal of the platform were then drawn, followed by the creation of a mid-fidelity prototype in Figma [45]. (8) The logo for the platform was selected by the research team. Finally, the high-fidelity clickable prototype was designed in Figma. (9) The prototype and their archives were delivered to a digital engineer for the development of the platform. Due to the length of the manuscript for and the objective of this study, namely, presenting the protocol of the study, the UX process will be presented in detail in further articles.

The “Personal COVID” intervention is being implemented through a responsive web application. This type of system can adapt to different screen sizes and resolutions, such as computers, tablets, and cellphones. This tool adapts the page design, resizes images or cuts them proportionally [44,46].

The content of the modules was created by mental health experts. Then, a clinical psychologist narrated the modules, and in parallel, the design team, composed of ten graphic designers, designed the videos that were adapted to the cultural and usual language of the Mexican population.

#### 2.5.2. Description of the Intervention

The “Personal COVID” platform is an online multi-component intervention that is aimed at healthcare workers that will be applied in two ways: video and text. The platform is available on the website www.personalcovid.com, accessed on 24 September 2022. “Personal COVID” consists of nine core modules and three complementary ones, which aim to reduce anxiety and depression symptoms, burnout, and compassion fatigue and to increase quality of life, sleep quality, resilience, and self-care. Each session will be available every three days. Participants will have the option of taking the three complementary modules at the end of the nine modules. The content of the sessions can be consulted in Table 2.

**Figure 3:**
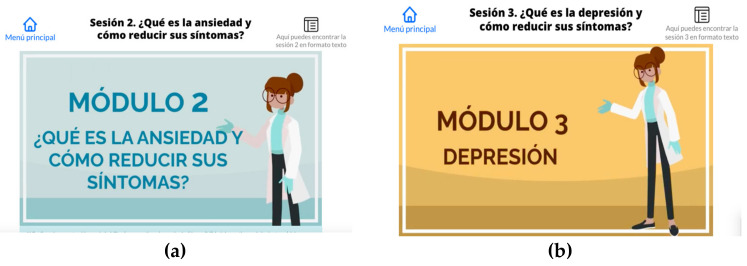
Animated videos of anxiety and depression sessions. (**a**) anxiety module; (**b**) depression module.

The content of each module can be seen through an animated video. An avatar appears in each video that explains the topic and each of the activities proposed for the participants (Figure 3). As mentioned above, all the content of each of the videos was created by expert psychologists.

#### 2.5.3. Active Control Group

The active control group consists of the implementation of the same contents of the self-applied intervention but implemented by psychotherapists through videoconference. The therapist will contact the participant via email, through an email with the domain *“Personal* *COVID*”, so the participant is aware of being a member of the project. The periodicity of the sessions will be the same as the deliberate intervention on the platform (each three days). Related to the confidentiality of each participant in the active control group, each of them will be assigned a code, which will be made up of their initials plus 3 letters, plus the date (month, day of the year), plus the corresponding university of the therapist, for example UNAM or UDG.

##### Training of the Therapists of the Online Treatment

The therapists of the active control group are five from the National Autonomous University of Mexico and five from the University of Guadalajara, supervised by EBV and ADLG at the first institution and RJMA on the second one. Therapists are psychologists with experience in clinical practice; some of them are students in a master’s degree course in health psychology. All the therapists will receive prior training on the “Personal COVID” intervention. The therapists were informed about the study design and the detailed contents of the intervention were shared with them through a manual prepared by the researchers. Afterwards, another online session was offered in order to clarify any doubts that the therapist may have regarding the materials and the procedure to provide the treatment to the participants. The supervisors will coordinate the logistics of work between the therapists; they will also review the progress of the therapists with the participants and attend to any situation that requires immediate attention. For further monitoring of the therapists, constant communication will be maintained. In addition, each therapist will deliver a report per session. It should be noted that the supervisors have clinical experience in the implementation of teletherapy; likewise, they have the knowledge to design the study and the intervention program that will be applied in both modalities.

### 2.6. Description of “Personal COVID” Platform

The platform includes a main page including the sections of (1) “team”, where all the team members that compose the project are presented; (2) “about” that provides an explanation regarding the objective of the platform; (3) “Frequently Asked Questions”, with extra information that could help the participant to know more about the intervention and its objectives; (4) “my account” and (5) “register”, that allows the participant to create an account.

**Figure 4:**
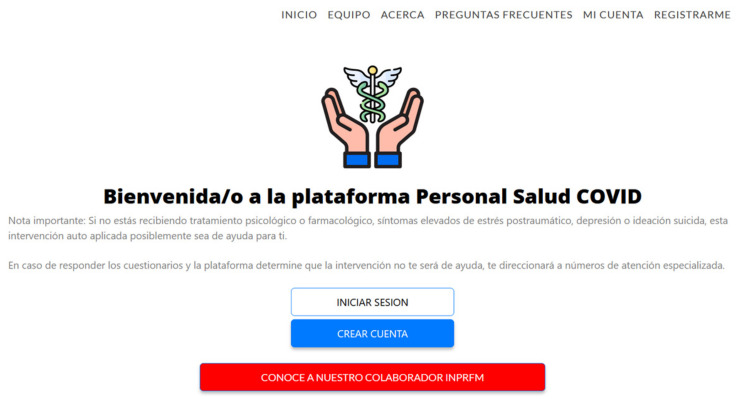
Main page of the Personal COVID Intervention where the participants need to log in (“iniciar sesion”) or create an account (“crear cuenta”).

The “my account” option provides the option to reset the password in case that is necessary (Figure 4).

**Figure 5:**
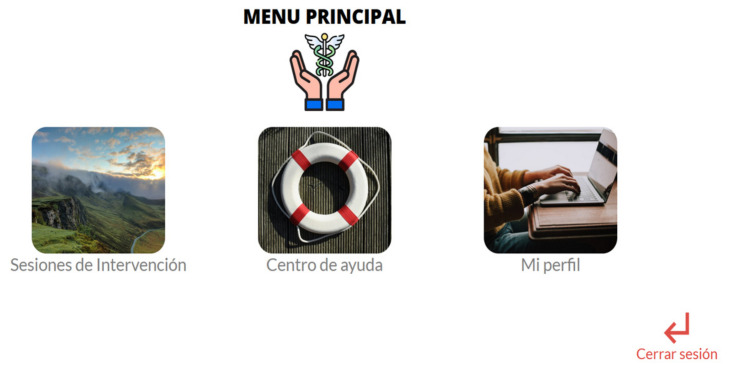
Main menu of the Personal COVID Intervention with the onboarding process indicating to the participant how to use the platform.

On the main page also appears the company that developed the platform (ITLAB), and from here it is possible to access the privacy policy and download the approval of the ethics committee of the Autonomous University of Juarez City. Once participants create an account, they are requested to read and accept the informed consent in order to proceed and fill the questionnaires. If all the inclusion criteria are fulfilled by an applicant, and no exclusion criteria are applicable, then the platform sends an email that the participant has registered properly and soon will be contacted in order to proceed. In this step and with the permuted blocks randomization, the participant is assigned to the self-applied intervention (experimental group) or the treatment with the therapist (active control group). For the participants that receive the self-applied treatment, the first time that they have access to the intervention, the platform provides an onboarding process (brief tutorial) with the main functions of the system. After the onboarding process, the participant accesses the main menu that includes 3 options: (1) Intervention sessions, (2) Help center, and (3) My profile (Figure 5).

**Figure 6:**
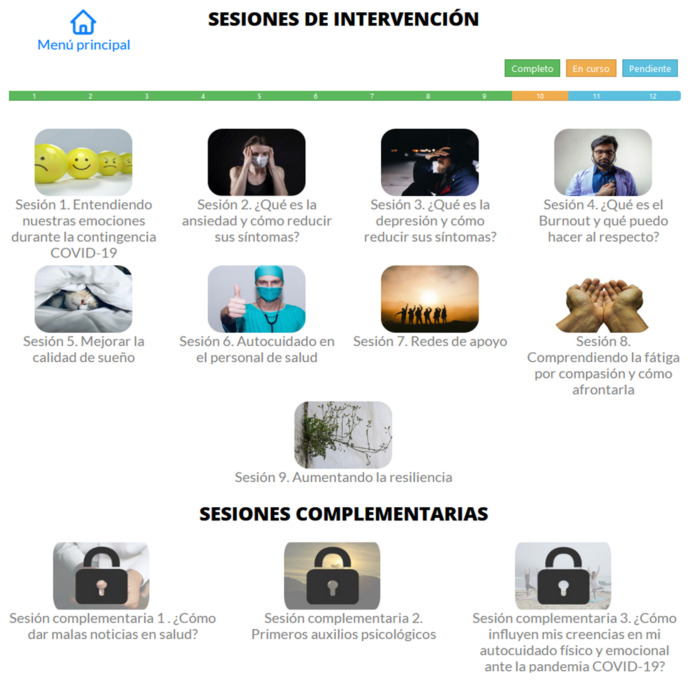
Screen with all the available sessions or modules.

On the first option, the participant can access the intervention sessions in video or text format as the participant prefers. This section also offers a bar status that indicates the modules already completed, those that are in process and those that are pending (Figure 6).

Under the second option, the participant can access the section of frequently asked questions that will be updated constantly, and also an option that offers the possibility to contact institutions for psychological support and an option to report technical problems. On the third option, the participant can observe the already done sessions, to adjust how many emails he/she wants to receive between sessions (1, 2 or 3), and the settings where the participant can see his/her details, such as the email and the option to modify the password and the status about the sessions. The participants who will be in the self-applied group will receive an email between sessions with the relevant information of the upcoming session. For example, if they are on module 4, the participant will receive an advance notification of the content of module 5 via email. This is intended, first, to fulfill what the participants request; and, second to attempt to reduce the dropout rate.

#### Technical Details of the Platform

The web-based system was developed in Visual Basic language, NET LANGUAGE. This allows designers and developers: to separate the parts of the system, to achieve better code management, to use more secure techniques and to have an efficient development. In this sense, the platform is the result of data structures and classes that are linked in a three-layer structure. The visualization layer is managed through HTML, and communicates with the “business rules” layer, which in turn manages the interaction with the data layer.

The development was carried out with the Git version manager, and the continuous integration and continuous delivery methodology was used. This allows the code quality to be adequate and the environment to be available via the internet.

### 2.7. Data Collection

The data will be collected through standardized questionnaires included in the platform www.personalcovid.com, accessed on 24 September 2022. The instruments will be answered in four moments of the study: (a) prior to entering the platform, and (b) at the end of all modules, (c) three months and (d) six months after the intervention. Participants will not receive any financial compensation for their participation. The data will be stored securely, participants cannot be identified, and the data will only be accessible to the person in charge of processing and analyzing the data.

#### 2.7.1. Primary Outcomes

##### Sociodemographic Data

Sociodemographic data will be collected such as schooling, employment status, if the person works where s/he works (e.g., public hospital, private hospital), the role in the hospital, years of experience, age, gender, city where the participant lives, and substance use or if the participants is undergoing psychological or psychopharmacological treatment, in order to assess compliance with the inclusion and exclusion criteria.

##### Scale for Generalized Anxiety Disorder (GAD-7)

The Spanish version of García-Campayo et al. [47] will be used. The GAD-7 is a 7-item scale designed to assess the severity of symptoms of generalized anxiety disorder. Answers are based on symptoms perceived in the past week. A score between 0 and 4 points indicates that anxiety is not perceived, and a score between 15 and 21 is an indicator of severe perceived anxiety [48]. This scale has good reliability (Cronbach’s alpha of 0.94) [47].

##### Center for Epidemiologic Studies Depression Scale, Revised Version (CES-D-R)

The CES-D-R is a self-report scale that assesses the symptoms of depression based on the criteria of temporality (in the past week or last two weeks) established in the DSM-5. It consists of 35 questions that contain 5 possible answers: barely (0 to 1 days), sometimes (1 to 2 days), occasionally (3 to 4 days), most (5 to 7 days) and almost daily (from 10 to 14 days). It has been shown to be a reliable scale in people of Mexican nationality (Cronbach’s alpha of 0.93) [49].

##### Professional Quality of Life Measure (ProQol-V)

The ProQOL scale measures quality of life of workers in caring or helping professions [50] through 30 items which are answered in a six-point Likert-style scale, ranging from 0 (Never) to 5 (Always). The instrument has shown adequate reliability for burnout (Cronbach’s alpha of 0.80), secondary traumatic stress (Cronbach’s alpha of 0.84), and compassion satisfaction (Cronbach’s alpha of 0.90) scales [51].

##### The Pittsburgh Sleep Quality Inventory (PSQI)

The PSQI [52] assesses the quality of sleep through 19 items that are grouped into 7 components (subjective sleep quality, sleep latency, duration of sleep, habitual sleep efficiency, sleep disturbances, use of sleep medications, and daytime dysfunction). These items are scored on a scale of zero to three. This instrument has proven to be reliable (Cronbach’s alpha of 0.78) [53].

##### Scale for Measuring Resilience in Mexicans (RESI-M)

This instrument is composed of 43 items with four response options: totally disagree, disagree, agree and totally agree. The items are grouped into 5 dimensions: (1) Strength and self-confidence, (2) Social competence, (3) Family support, (4) Social support and (5) Structure. The instrument was determined to have a high degree of reliability (Cronbach’s alpha of 0.93) and validity [54].

##### Self-Care Skills Scale (SKS)

The SKS scale developed by Evers et al. [55], and the version validated in Mexico by Gallegos [56] will be applied. The CAC is composed of 24 Likert-type items with four types of response (from one to four). By adding all the items, a score of 24–96 can be obtained, indicating higher values, and greater capacity for self-care [57]. The validity and reliability of the scale in Spanish in the Mexican population was adequate (Cronbach’s alpha of 0.77) [57].

#### 2.7.2. Secondary Outcomes

##### Post-Traumatic Stress Scale (PTSD)

The version validated into Spanish will be used [58], which consists of 17 items and classifies the severity of PTSD symptoms in the last two weeks. The total severity score is calculated through the sum of all the items and can range from 0 (total absence of symptoms) to 51 (severely affected). The diagnosis is made when one symptom of re-experiencing is observed, three of avoidance and two of activation [59]. The instrument has shown adequate psychometric properties (the subscales that compose the instrument obtained Cronbach’s alpha equal to or greater than 0.90).

##### Plutchik Suicide Risk Scale (PSRS)

This questionnaire assesses the risk of suicide with questions with dichotomous answers (yes/no), that explore the history of suicide attempts, suicidal ideation, and suicide plans. This scale establishes a cut-off point of >6 that differentiates people at risk from those who are not at risk of suicide [60]. This scale has shown good reliability properties (Cronbach’s alpha of 0.74) and has been used in studies with the Mexican population [61].

##### Fear of COVID-19 Scale (FCV-19S)

The FCV-19S was created by Ahorsu et al. [62]. It is a short scale that assesses fear of the COVID-19 disease, and is made up of seven Likert-type items, with ratings ranging from 1 (totally disagree) to 5 (totally agree), so the final scores range from 7 to 35 points. The higher these scores, the greater the fear of COVID-19. This scale was shown to have a high degree of reliability (Cronbach’s alpha of 0.82). The version in Spanish of this scale validated by Huarcaya-Victoria et al. [63] will be applied.

##### Self-Assessment by Module

At the end of each of the modules, a self-assessment will be carried out, in which self-exploration questions will be asked related to the information that was reviewed throughout the module sessions, in order to probe how the healthcare personnel apply the knowledge acquired.

##### Opinion Treatment

This questionnaire consists of four questions that report the level of satisfaction with the treatment: if they would recommend the treatment to a friend or relative, if the patient considers the treatment useful, and if they think that the treatment was difficult to handle or if it was aversive. The items are answered on a scale from 1 (not at all) to 10 (a lot) [64].

##### System Usability Scale

This is an instrument designed to validate the usability of a system. It is composed of 10 items, which are answered on a five-point Likert-type scale with respect to the degree of comfort of the product, ranging from 1 (totally disagree) to 5 (completely agree). To obtain the global score of this scale, all the values obtained must be added and multiplied by 2.5, and this will result in a number between 0 and 125, which will be the global value on this scale [65].

### 2.8. Data Analysis

The data from this study will be analyzed using the program SPSS v.26. A composition of data monitoring committee (DMC) is not necessary since two of the authors of this study will have access to the participant information that is automatically saved on the platform to monitor the participation in the platform. Only participants who complete all sessions of the intervention will be included. Shapiro–Wilk normality tests, descriptive statistics, and *t*-tests for independent samples will be carried out to analyze the basic differences between groups, when the assumption of homogeneity of variance is fulfilled, and Cohen’s d to calculate the effect size (ES) in these cases. The Mann–Whitney U test and Rosenthal’s r will be used when the assumption of homogeneity of variance is not fulfilled. According to Cohen, values ≈ 0.20 indicate a small ES, medium ≈ 0.50, and high ≈ 0.80.

In addition to the above, after checking the assumptions, a simple ANCOVA will be performed between groups, with two treatment conditions—experimental group and active control group—to observe if there is an effect on anxiety and depressive symptoms, burnout, compassion fatigue, quality of life, sleep quality, resilience, and self-care. The covariate will be the pre-intervention score. The partial eta squared will be used to evaluate the effect size, knowing that the values ≈ 0.02 indicate a small effect size, ≈ 0.15 medium and ≈ 0.30 large.

#### Validity and Reliability/Rigour

This research has a research design considered as the highest quality benchmark in terms of experimental research to evaluate the effectiveness of a treatment [66]. Widely used instruments that have adequate psychometric properties will be administered in the evaluation of the participants. Moreover, the statistical analysis that will be carried out will be adequate for the present research design. In addition to the above, trial participants, outcome assessors, and research data analysts will be blinded to the allocation of participants to the intervention groups, in order to reduce biases in the assessment of the effects of the intervention. The study design, procedures and reports will follow the recommendations of the SPIRIT statement [67].

### 2.9. Ethical Considerations

This study has obtained the approval of the Research Ethics Committee of the Autonomous University of Juarez City (CEI- Ref No: CEI-2021-1-09) in March 2021 and is registered in Clinical Trials (NCT04890665).

#### Informed Consent

For admission to the intervention in both groups (experimental and control), the participants must read and accept the informed consent, where they will be informed about the objective of the study, the evaluation instruments, the participation options, the privacy policy, and their rights. In the same way, they will be informed about the possible referral to another institution where they can be treated in the case of having a high score in post-traumatic stress disorder or depressive disorder or presenting suicidal ideation or previous suicide attempt (Document S2, Supplementary Materials). The participant may withdraw their authorization whenever they deem it appropriate, at any point in the process. The research team will be available to answer questions at any time. All data collected will be pseudo-anonymous and will be kept confidential.

## 3. Discussion

In the present study, the process of developing a multi-component psychological intervention for healthcare workers in Mexico is presented, aimed at treating emotional disorders, and promoting health. The COVID-19 pandemic constitutes a new and a general social health challenge, where healthcare workers are especially affected, presenting an increase in emotional symptoms, stress, burnout, and compassion fatigue [5,6].

In addition to psychological discomfort, the positive capacities of healthcare workers have also been evaluated and supported in various studies, since they help reduce the emotional impact and improve coping strategies (resilience) that promote well-being, self-care, and improvement in quality of life [17,19,20,22,24]. Healthcare workers’ well-being will have a potential impact on their capacity for taking care of patients who may be infected by the COVID-19, consistent with the evidence suggesting that being burned out at work reduces the quality of the health assistance.

High levels of resilience, coping strategies and social support have been found. Some of the most used coping strategies are seeking support with family and colleagues, as well as religion [68], which have been associated with lower levels of burnout, emotional exhaustion, and compassion fatigue in healthcare workers [6]. Being older, looking for information on mental health, having a higher educational level, lower levels of depression and social dysfunction, are some of the predictors of resilience that have been found in health personnel [69]. Thus, there arises the need to develop and implement psychological intervention programs to promote the comprehensive well-being of this population. Digital psychological interventions have been a viable option to support healthcare workers during the COVID-19 pandemic. In addition, they have shown high acceptance and satisfaction by healthcare workers. Some of the implementation modalities are the use of a platform, telephone support, short videos, and even online games [19].

The modules of the platform “Personal COVID” will be designed taking into account theoretical perspectives of psychology, which have been shown to be effective for the treatment of psychological problems, such as Cognitive Behavioral Therapy, Acceptance and Commitment Therapy, Mindfulness, Positive Psychology, and the Health Belief Model [19,70,71,72,73].

Our protocol was designed according to the highest scientific standards, so it is expected that it can lay the foundations for the care of healthcare workers, and that it can help to make social and political decisions that are aimed at treating this group psychologically. With our design, we could also analyze if it is possible to obtain similar results through a self-applied intervention and through exactly the same contents provided by a therapist. If the results are positive, in terms of finding similar efficacy rates between the conditions, and similar drop-out rate, then the study could provide more data on how to reduce the abandoning of self-applied treatments by applying UX resources.

In addition, a self-applied online intervention could reduce costs, both in treatment for patients and in payments for days of disability for companies or government agencies. Such an intervention could reach a greater number of people, to meet the broad mental health needs that have been detected in the COVID-19 pandemic, mainly in Latin America, where even before the pandemic there was already a treatment gap for mental disorders [74]. In the systematic review by Hedman et al. [75], the authors conclude that the literature regarding cost effectiveness is scarce, but the most probable estimation is that internet-based CBT is a cost-effective intervention compared with no treatment. The results that will emerge from Personal COVID would be relevant to expand the literature in online treatments in Latin America, and how self-applied interventions could enable more evidence-based psychological interventions to be provided to health care workers and the general population.

The treatment platform is designed for being user-friendly, so it could be easily used by the subjects with a relative independence of their internet expertise. Finally, the results of the present study could provide evidence about the most suitable psychological treatment for healthcare workers facing COVID-19. This intervention protocol contemplates a multi-component model that, in accordance with other psychological interventions, not only seeks to mitigate the emotional impact but also seeks to promote positive domains in health personnel [20]. As a result of this, the intervention could help to prevent the psychopathological issues that have been noted all over the world, significantly reducing the emotional suffering of those who coped with the critical medical situations of the global COVID-19 health crisis before the onset of a mental disorder diagnosis.

## 4. Limitations

Despite the strengths of our work, it also has limitations. Among the main limitations of the study is the fact that it did not have an active control group, which could offer information about the efficacy of the treatment. However, for ethical reasons it is not appropriate to leave the participants without receiving the intervention, as this could have a negative impact on their symptoms, which could be in line with a nocebo effect [76].

Furthermore, we have a digital platform designed for taking into account the principles of UX, so that in future research, we could compare our intervention with another that is not based on these principles. In this way, we could evaluate if the UX process generates greater adherence and better health results. Due to the available budget to carry out this intervention, and the urgency of having it available to attend to the emergency during the COVID-19 pandemic, this aspect has not been currently addressed. However, it may be considered in future studies in situations less adverse, where it is possible to make such a comparison without involving the risk of the life of third parties, in this case the patients attended by healthcare personnel.

The final limitation refers to the nature of the instruments, which will be self-applied, and which may affect the responses provided, if the participants do not clearly identify their symptoms. It has been seen with this population that the expression of emotions is perceived as unprofessional because they must handle unexpected emotions that emerge in themselves and from patients [77]. This, of course, has affected their mental health and may affect their adherence to psychological treatments. For this reason, it is expected that the group that will receive the intervention in videoconference format will be able to verify the symptoms provided in the instruments through the perception of the therapists. However, in the group that will receive the care entirely through the platform, it will not be possible to verify that participants have identified their symptoms. In this regard, work is being done on systems to evaluate responses in a more objective way through technologies such as virtual reality, portable sensors, telephones, and biofeedback techniques that can be very useful for this objective [78]. Nonetheless, in this study the aforementioned technologies will not be applied.

## 5. Conclusions

It is important to provide digital psychological interventions to healthcare workers, given the psychological impact they have experienced since the start of the COVID-19 pandemic. The modality of our intervention, through a self-applicable platform, can bring advantages, such as greater anonymity, and reach a greater number of participants. In addition, this study will provide scientific evidence regarding the efficacy of the intervention since we will follow the recommended quality standards. Once its effectiveness has been shown, a similar program might be offered to all health workers in México during COVID-19 or other health crisis.

## Figures and Tables

**Table 1:** Schedule of enrolment, interventions, and assessments according to the SPIRIT guidelines.

	Study Period						
	Enrolment	Allocation	Post-Allocation				
Timepoint	*t*_1_	0	Pre Test	Intervention	Post Test	Follow-Up-3	Follow-Up-6
**Enrolment**							
Eligibility screen	X						
Informed consent	X						
Allocation		X					
**Interventions**							
Personal COVID intervention (experimental group)			X	X	X	X	X
Intervention with therapists (control group)			X	X	X	X	X
**Assessments**							
**Primary outcomes**							
Sociodemographic data	X						
Scale for Generalized Anxiety Disorder			X		X	X	X
Center for Epidemiologic Studies Depression Scale, revised version	X				X	X	X
Professional Quality of Life Measure			X		X	X	X
The Pittsburgh Sleep Quality Inventory			X		X	X	X
Scale for measuring resilience in Mexicans			X		X	X	X
Self-care skills scale			X		X	X	X
**Secondary outcomes**							
Post-traumatic stress scale	X				X	X	X
Plutchik Suicide Risk Scale	X				X	X	X
Fear of COVID-19 Scale	X						
Self-assessment by module				X			
Opinion treatment					X		
System Usability Scale					X		

**Table 2:** Contents of the intervention modules.

Module	Objectives	Theoretical Model	Description
1. Understand our emotions during the COVID-19 contingency.	Learn about the importance and adaptive goals of anxiety symptoms and emotions.	CBT ^1^	Psychoeducation will be offered to the participants about what emotions are, and why we need them, highlighting the importance of emotional regulation as a fundamental factor for mental health.
2. What is anxiety and how to reduce its symptoms?	Know what anxiety is, its symptoms and the situations that generate it.	CBT ^1^	Psychoeducation will be provided on what anxiety is, what are its symptoms and associated experiences. The thought-stopping technique is explained, and its use will be modeled in the face of anxiety symptoms. Finally, information will be provided on pleasant day-to-day activities that can reduce anxiety symptoms.
3. What is depression and how to reduce its symptoms	Provide information and tools to manage depressive moods, through a multi-component approach.	CBT ^1^ and BA ^2^	The multi-component approach will includes: (a) self-observation and identification of vicious cycles of depressive moods, related to the loss of positive reinforcements in daily life; (b) the progressive reincorporation of rewarding events, considering pleasantness and cost/effort of such activities, (c) the role of sabotaging thoughts in maintaining vicious circles.
4. What is burnout and what can I do about it?	Know the concept of burnout, its symptoms, and strategies to reduce it.	CBT ^1^	The symptoms and causes of burnout will be shown as well as strategies for its reduction, such as the programming of pleasant activities, detection of thoughts and emotions related to workload.
5. Improve sleep quality	Increase time and sleep quality by a multidimensional intervention.	BBII ^3^ and CBT-I ^4^	Multidimensional intervention that includes: sleep restriction, stimulus control, sleep hygiene, muscle relaxation, and psychoeducation related to the impact of irrational thoughts on the sleep pattern.
6. Physical and emotional self-care	Psychoeducate on general measures of physical/emotional self-care, and character strengths.	CBT ^1^ and PP ^5^	General recommendations for physical/emotional self-care are given, as well as the main personal strengths of character.
7. Social support networks	Encourage contact with support networks in order to reduce stress in difficult stages.	CBT ^1^	The importance of maintaining contact with a social support network to express emotions and be able to exchange experiences will be highlighted.
8. How do you deal with compassion fatigue?	Identify what compassion fatigue is, the reasons why it occurs, and how to reduce it.	Mindfulness and ACT ^6^	Understand what compassion fatigue is and how it may be affecting your performance and personal life during the pandemic, as well as providing tools to cope with it.
9. Increasing resilience	(a) Recognize personal capacities to recover after a stressful event.	CBT ^1^	The importance of promoting resilience will be shown, as it helps us face critical situations in a healthy and enriching way.
**Complementary modules**			
1c. How to give bad news related to health?	Train in how to deliver bad health news, based on the SPIKES protocol.	SPIKES protocol	The 6 steps proposed by the SPIKES protocol to give bad health news are described, and practical cases will be presented.
2c. Psychological first aid (how to deal with patients that are in an anxiety crisis).	(a) To know and understand what these crises are. (b) To know and apply psychological first aid.		Information is provided about the crisis and their management through the PAP, with the objective of being applied to patients of healthcare workers in Mexico.
3c. How do my beliefs influence my physical and emotional self-care in view of the COVID-19 epidemic?	Psychoeducate about the HBM component.	HBM ^7^	Information will be provided about the types of beliefs and the external factors that influence the adoption of healthy behaviors.

^1^ Cognitive Behavioral Therapy; ^2^ Behavioral Activation; ^3^ Brief Behavioral Intervention for Insomnia; ^4^ Cognitive Behavioral Therapy for Insomnia; ^5^ Positive Psychology; ^6^ Acceptance and Commitment Therapy; ^7^ Health Belief Model.

## Data Availability

Not applicable.

## Supplementary Materials

The following supporting information can be downloaded at: https://www.mdpi.com/article/10.3390/ijerph191912749/s1, Table S1: SPIRIT 2013 Checklist: Recommended items to address in a clinical trial protocol and related documents; Document S2: Cover page with the Official Title of the study: Effectiveness of a Self-applied Multi-component Psychological Online Intervention Based on UX, for the Reduction of Anxiety, Depression, Burnout, Stress, Fatigue Compassion, and Increase in Quality of Life, Sleep Quality and Self-care on Healthcare Workers During the COVID-19 Outbreak: A Randomized Clinical Trial.
