# Supplementary material for: E-Health Psychological Intervention for COVID-19 Healthcare Workers: Protocol for its Implementation and Evaluation
Source: Int J Environ Res Public Health. 2022 Oct 5;19(19):12749. doi: 10.3390/ijerph191912749 (PMC9566813; doi:10.3390/ijerph191912749)
Supplement: Supplementary file 1 [file ijerph-19-12749-s001.zip › ijerph-1901794-supplementary-Document S2.pdf]

Cover page with the Official Title of the study:
Effectiveness of a Self-applied Multi-component Psychological Online Intervention Based on UX, for the Reduction of Anxiety, Depression, Burnout, Stress, Fatigue Compassion, and Increase in Quality of Life, Sleep Quality and Self-care on Healthcare Workers During the COVID-19 Outbreak: A Randomized Clinical Trial

Date: April 22nd, 2021

UNIVERSIDAD AUTONOMA DE CIUDAD JUAREZ

viu Universidad Internacional de Valencia

UNIVERSIDAD NACIONAL AUTONOMA DE MEXICO

UNIVERSIDAD DE GUADALAJARA

UNIVERSIDAD DE BUENOS AIRES

UIDE
Universidad Internacional del Ecuador

INFORMED CONSENT

A group of researchers from the International University of Valencia (VIU), the Autonomous University of Ciudad Juárez (UACJ),the Autonomous University of Mexico (UNAM), the University of Guadalajara (UDEG), Universidad Internacional del Ecuador (UIDE), and the University of Buenos Aires (UBA) Argentina, we are conducting a study in order to implement and validate an online intervention based on Cognitive Behavioral Therapy, Mindfulness, Behavioral Activation Therapy, Acceptance and Commitment Therapy, and Positive Psychology with the purpose of providing a psychological treatment for healthcare workers from private or public hospitals in México in order to reduce the symptoms of anxiety, depression, burnout, and post-traumatic stress, and increasing the quality of sleep and perception of the quality of life.

The intervention is composed by 9 interconnected nuclear modules and optional modules to which you will have access when registering on the platform.

To register on the platform, only an email is required, and you do not have to provide sensitive or personally identifiable information such as your name at any time. The results of this research will be analyzed for research purposes and, therefore, they can be published in journals, scientific books or disseminated by other means to the scientific community, taking care at all times of the confidentiality and anonymity of the data of the participants. This study has the approval of the Research Ethics Committee, from the Universidad Autónoma de Ciudad Juárez. Approval date April 22nd, 2021. Identification number: CEI-2021-1-332. It is important to mention that you will not be asked for any sensitive data or your name, in addition to the fact that the intervention will always be completely free.

For this reason, to start we ask you to answer a series of questions that will be presented below, a task that will take you around 40-45 minutes. If there is a question that you do not understand, please report it to us and continue with the next question, the important thing is that you answer what exactly happens in your case, because that is precisely what we want to know. There are no right or wrong questions, so please answer with the utmost sincerity.

The instruments to answer are:

1. Post-traumatic diagnostic scale (PDS)
2. Beck Scale for Suicide Ideation (BSS)
3. Generalized Anxiety Scale (GAD-7).
4. The Center for Epidemiologic Studies Depression Scale-Revised (CESD-R)

5. Professional Quality of Life Measure (ProQOL)
6. Pittsburgh Sleep Quality Index (PSQI)
7. Scale for measuring resilience with Mexicans (RESI-M)
8. Appraisal of Self-care Agency (ASA)
9. The Fear of COVID-19 Scale (FCV-19S)
10. Opinion on the treatment.
11. System usability scale (SUS)

If you agree with what is proposed, please click on accept, otherwise you can close your internet window. In this application, you indicate that you agree to voluntarily participate in this research that we are conducting and, therefore, willing/complete the questionnaires.
I accept and commit to respect the following standards in order to provide knowledge, progress, and research on the health of the Mexican population that is being exposed to the contingency due to the COVID-19 pandemic, therefore, I declare that:
• I have been able to ask questions about the study
•I have received enough information about the study
• I can contact the people who are applying this series of questionnaires to dispel my doubts regarding the research.
I understand that my participation is voluntary and that I can withdraw from the study:
1. Anytime
2. Without having to explain
3. Without this affecting my integrity as a person I freely agree to participate in the study
Benefits:
If you accept your participation, you will receive a Psychological intervention, based on scientific evidence and completely free at all times, through a program of 12 interactive modules.
For more questions, you can contact us by mail at admin@saludmentalcovid.com or Dr. Alejandro Domínguez Rodríguez, principal investigator, and coordinator of this project: alejandro.dominguez.r@campusviu.es

I freely agree to participate in the study:

Date:
